# Vestibular migraine without headache treated with lomerizine: A 35‐year‐old woman undiagnosed for 10 years

**DOI:** 10.1002/jgf2.311

**Published:** 2020-03-11

**Authors:** Hiroki Maita, Tadashi Kobayashi, Takashi Akimoto, Hiroshi Osawa, Hiroyuki Kato

**Affiliations:** ^1^ Development of Community Healthcare Hirosaki University Graduate School of Medicine Aomori Japan; ^2^ General Medicine Hirosaki University Graduate School of Medicine Aomori Japan; ^3^ Department of General Medicine Hirosaki University School of Medicine & Hospital Aomori Japan

**Keywords:** benign paroxysmal positional vertigo, lomerizine, Meniere's disease, migraine‐associated vertigo, vestibular migraine

## Abstract

A 35‐year‐old woman presented with recurrent vertigo without headache, which had persisted for 10 years. Detailed medical history revealed that she experienced hearing loss, tinnitus, nausea, photophobia, phonophobia, and slight discomfort in the head during vertigo attacks, which often led to absence from work. Based on the diagnostic criteria of the International Classification of Headache Disorders, third edition, she was diagnosed with vestibular migraine and was prescribed lomerizine, as prophylaxis. Her symptoms markedly improved, enabling her to go to work. Accurate diagnosis and treatment are important for improving the quality of life of patients, since vestibular migraine is commonly underdiagnosed.

## 1 INTRODUCTION

Vestibular migraine, also called migraine‐associated vertigo, is a relatively common (2.7% in the United States^1^, 4.6% in Japan^2^) and commonly underdiagnosed^1^, ^3^ disease, which was defined in 2012.^4^ Vestibular migraine is considered a variant of migraine and is characterized by vestibular symptoms, such as vertigo, dizziness, and balance impairment, and can present with or without a headache.^5^ The diagnosis of vestibular migraine presents great difficulty, especially in patients who complain of vestibular symptoms without headache. In Japan, lomerizine, a calcium channel blocker, is often used for migraine prophylaxis. However, few studies have reported its effectiveness in treating vestibular migraine^2^. Herein, we report a case of a woman with vestibular migraine without headache, which remained undiagnosed for 10 years. Lomerizine markedly improved her symptoms, preventing her from missing work.

## 2 CASE REPORT

A 35‐year‐old woman with a history of polycystic ovarian syndrome, glucose intolerance, and insomnia was referred to our hospital with a 10‐year history of recurrent vertigo without headache. She was treated based on a diagnosis of vertigo of unknown origin or benign paroxysmal positional vertigo, since no abnormalities were confirmed on head computed tomography (Figure S1), electroencephalogram, audiometry, and vestibular function tests conducted at other medical institutions. A detailed medical history revealed that her symptoms started with vertigo that occasionally worsened, which made walking difficult. The symptoms lasted for about half a day to one day, leading to absence from work three to four times a month. She also generally complained of hearing loss, tinnitus, nausea, photophobia, phonophobia, and slight head discomfort with her vertigo attacks, some of which were associated with migraine rather than vestibular disorders, such as Meniere's disease. Head computed tomography, auditory and vestibular function tests, and autonomic function tests revealed no abnormality in the remission period. Based on the diagnostic criteria of the International Classification of Headache Disorders, third edition,^6^ she was diagnosed with vestibular migraine and was prescribed lomerizine 5 mg twice daily as prophylaxis. Rizatriptan and metoclopramide were administered as acute treatment. A few days later, the frequency of dizziness attacks decreased by half. The duration of the attacks decreased to 0.5‐2 hours and the severity improved from 10 to 3 on the 11‐point numeric rating scale. Further, she did not experience any severe attacks 3 months after the prophylactic treatment. Mild attacks were controlled by self‐medication with rizatriptan and metoclopramide as needed. Moreover, she did not have to miss work, following the prophylactic treatment.

## 3 DISCUSSION

Our case study revealed the following: Vestibular migraine can remain undiagnosed for an extended period of time, detailed evaluation of medical history can contribute to the diagnosis, and lomerizine may be effective in preventing vestibular and common migraines.

Vestibular migraine without headache can easily remain undiagnosed for an extended period of time, unless confirmed by physicians, which usually entails obtaining a highly detailed medical history. The patient did not have a personal history or family history of medically confirmed migraine. We considered the repeated episodes of head discomfort consistent with migraine attack. It was reported that 64% of patients with vestibular migraine who had vertigo or dizziness did not present with headache at the time of their attack.^2^ Some surveys of the general population in the United States and Germany reported that only 10%‐21% of patients with vestibular migraine with or without headache, who met the criteria for vestibular migraine, were correctly diagnosed,^1^, ^3^ while the remaining patients were misdiagnosed with benign paroxysmal positional vertigo, Meniere's disease, anemia, diabetes, cervical vertigo, dehydration, and somatic symptoms during repeated episodes.^3^, ^5^ Moreover, in Japan, insufficient identification of the symptoms among physicians impedes proper diagnosis of vestibular migraine.^7^ Misdiagnosed patients are usually prescribed vestibular suppressants, instead of migraine‐specific treatments. In our case, the patient was diagnosed with vestibular migraine 10 years after the onset of symptoms. We consider that her medical history, including acute spontaneous vertigo which did not resolve at rest, persistent symptoms regardless of vestibular suppressants, and complete remission during the interval period, might be the key information that differentiates the case from other diseases.

Lomerizine, a calcium channel blocker, which selectively affects the cerebral circulation, may be effective in preventing vestibular and common migraines. Although the pathophysiology of vestibular migraine has not been fully elucidated, it might involve neuroanatomical pathways to and from central vestibular structures and neurochemical modulation via the locus coeruleus and raphe nuclei. In general, the treatment and prevention of vestibular migraine are adopted from those for common migraine (for acute treatment: rizatriptan, zolmitriptan,^8^ or metoclopramide;^9^ for preventive treatment: flunarizine, venlafaxine, or propranolol^10^). No large‐scale clinical trials have evaluated the effect of lomerizine, which belongs to the same class as flunarizine. We prescribed rizatriptan, metoclopramide, and lomerizine to our patient, and the severity and frequency of her symptoms improved remarkably. We consider the preventive treatment to have mainly contributed to the improvement of the severity of the disease.

Our patient's symptoms were quite severe that she considered leaving her job. According to reports, 40% of patients with vestibular migraine missed work because of their symptoms.^3^ Accurate diagnosis and treatment of vestibular migraine are crucial for decreasing the social burden of the disease and improving quality of life.

In conclusion, vestibular migraine without headache can easily remain undiagnosed for an extended period of time. Lomerizine may be effective in preventing vestibular and common migraines. Accurate diagnosis and treatment of vestibular migraine may improve health‐related quality‐of‐life outcomes in working‐age individuals.

## CONFLICT OF INTERESTS

The authors have stated explicitly that there are no conflicts of interest in connection with this article.

## Supporting information

Figure S1
