# Supplementary figures and images for: Vestibular migraine without headache treated with lomerizine: A 35‐year‐old woman undiagnosed for 10 years
Source: J Gen Fam Med. 2020 Mar 11;21(4):140–2. doi: 10.1002/jgf2.311 (PMC7388671; doi:10.1002/jgf2.311)

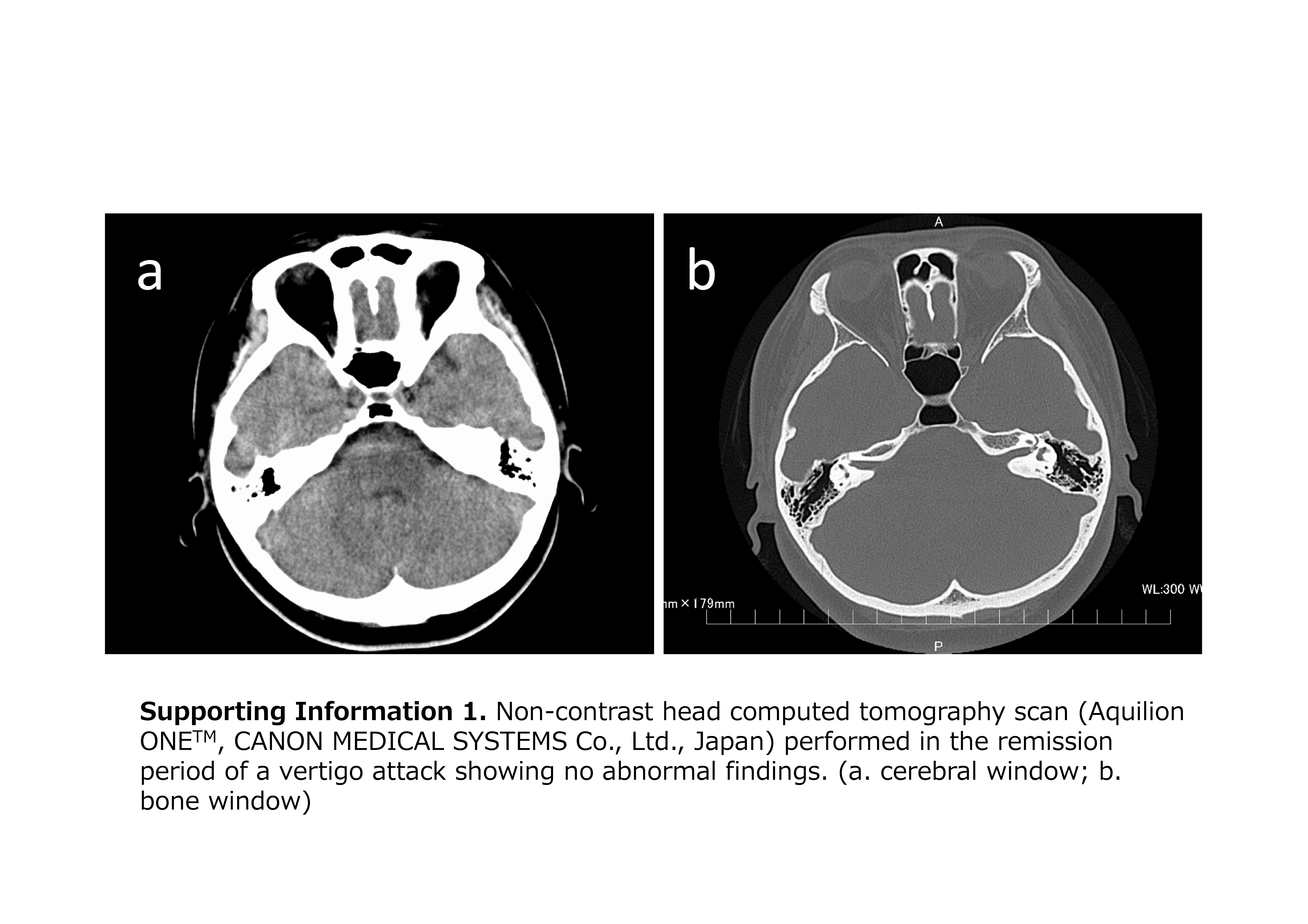

Supplement: Supplementary file 1 — Figure S1 [file JGF2-21-140-s001.tiff]
